# Supplementary figures and images for: Integrated bioinformatics analysis of potential pathway biomarkers using abnormal proteins in clubfoot
Source: PeerJ. 2020 Jan 20;8:e8422. doi: 10.7717/peerj.8422 (PMC6977474; doi:10.7717/peerj.8422)

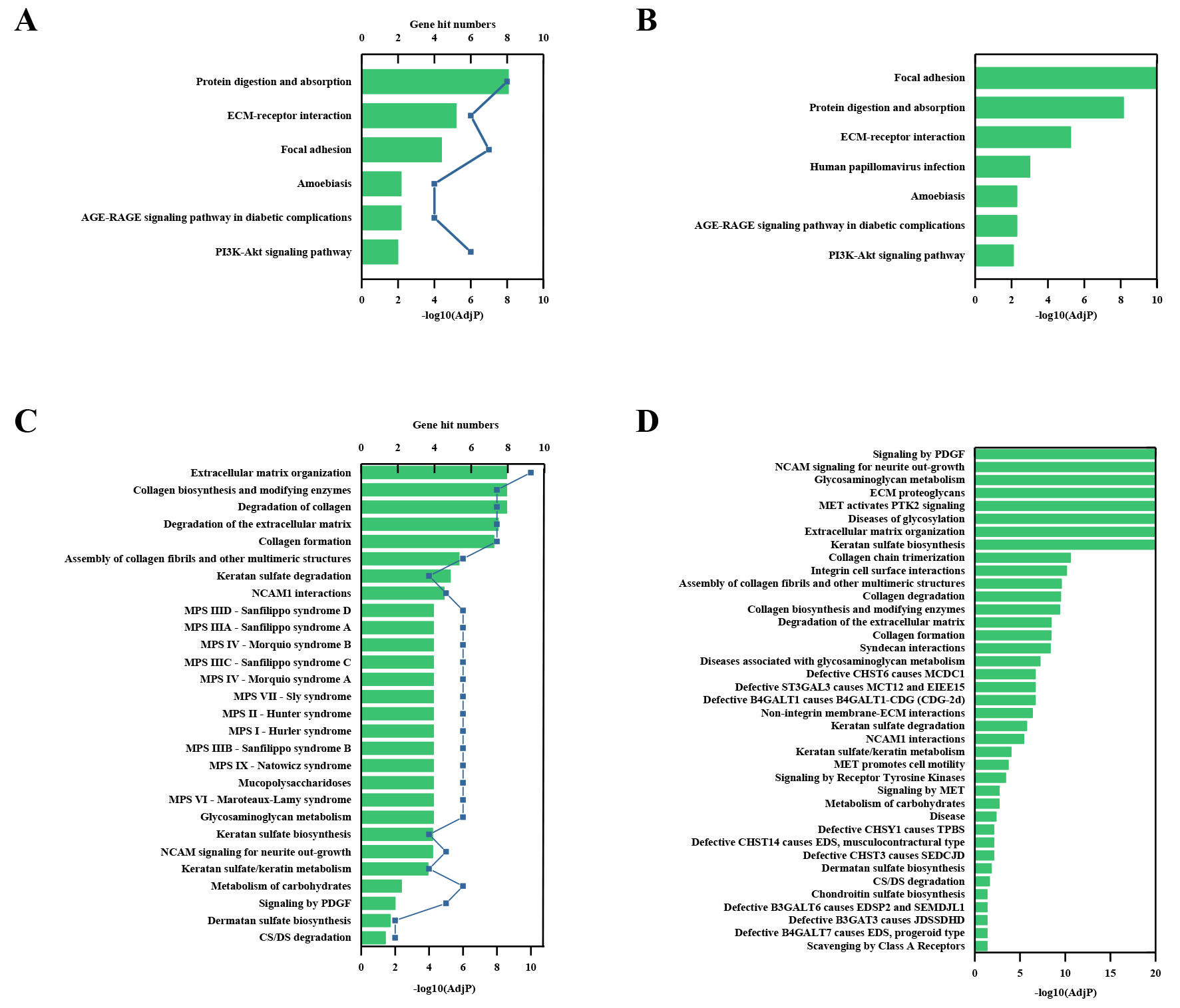

Supplement: Figure S1 — (A) KEGG pathway analysis by NetworkAnalyst. (B) KEGG pathway analysis by WebGestalt. (C) Reactome pathway analysis by NetworkAnalyst. (B) Reactome pathway analysis by WebGestalt. [file peerj-08-8422-s001.png]

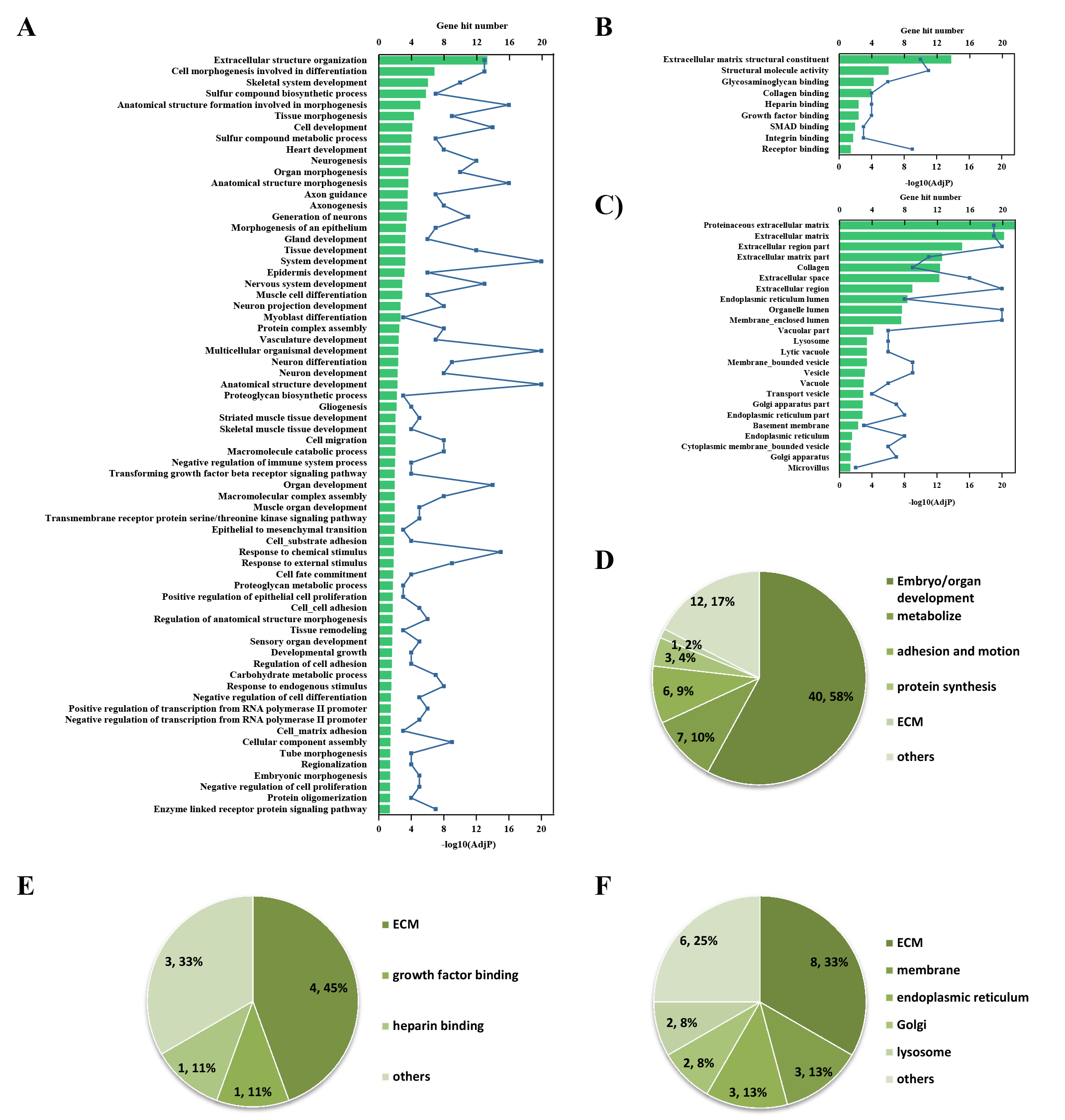

Supplement: Figure S2 — (A) (D) GO: BP. (B) (E) GO: MF. (C) (F) GO: CC. [file peerj-08-8422-s002.png]
